# Splenic injury from blunt trauma

**DOI:** 10.1093/bjs/znad060

**Published:** 2023-03-14

**Authors:** Johannes Wiik Larsen, Kenneth Thorsen, Kjetil Søreide

**Affiliations:** Department of Gastrointestinal Surgery, Stavanger University Hospital, Stavanger, Norway; SAFER Surgery, Stavanger University Hospital, Stavanger, Norway; STING, Stavanger Trauma Investigation Group, Stavanger University Hospital, Stavanger, Norway; Department of Clinical Medicine, University of Bergen, Bergen, Norway; Department of Gastrointestinal Surgery, Stavanger University Hospital, Stavanger, Norway; SAFER Surgery, Stavanger University Hospital, Stavanger, Norway; STING, Stavanger Trauma Investigation Group, Stavanger University Hospital, Stavanger, Norway; Department of Clinical Medicine, University of Bergen, Bergen, Norway; Section for Traumatology, Surgical Clinic, Stavanger University Hospital, Stavanger, Norway; Department of Gastrointestinal Surgery, Stavanger University Hospital, Stavanger, Norway; SAFER Surgery, Stavanger University Hospital, Stavanger, Norway; STING, Stavanger Trauma Investigation Group, Stavanger University Hospital, Stavanger, Norway; Department of Clinical Medicine, University of Bergen, Bergen, Norway

## Introduction

Injury to the spleen is one of the most common solid organ injuries in blunt trauma^1^. Following the primary and secondary survey in an injured patient, the potential dynamic alteration in the event of ongoing haemorrhage should be kept in mind. A sick patient should be in the operating theatre unless response to resuscitation and appropriate resources are available for simultaneous resuscitation and diagnostics (for example, a hybrid suite) that would allow rapid intervention. Hence, the response to any physiological challenge takes precedence, always. That said, cohort studies and large registries suggest that most isolated splenic injuries (up to 90 per cent) can be managed without an operation. However, angioembolization may play an important role in non-operative management. Concomitant injuries or risk factors may influence the success of non-surgical management. This article addresses some aspects in the evaluation and management of patients with splenic injury after blunt trauma.

## Severity scoring of the injury

**Fig. 1:**
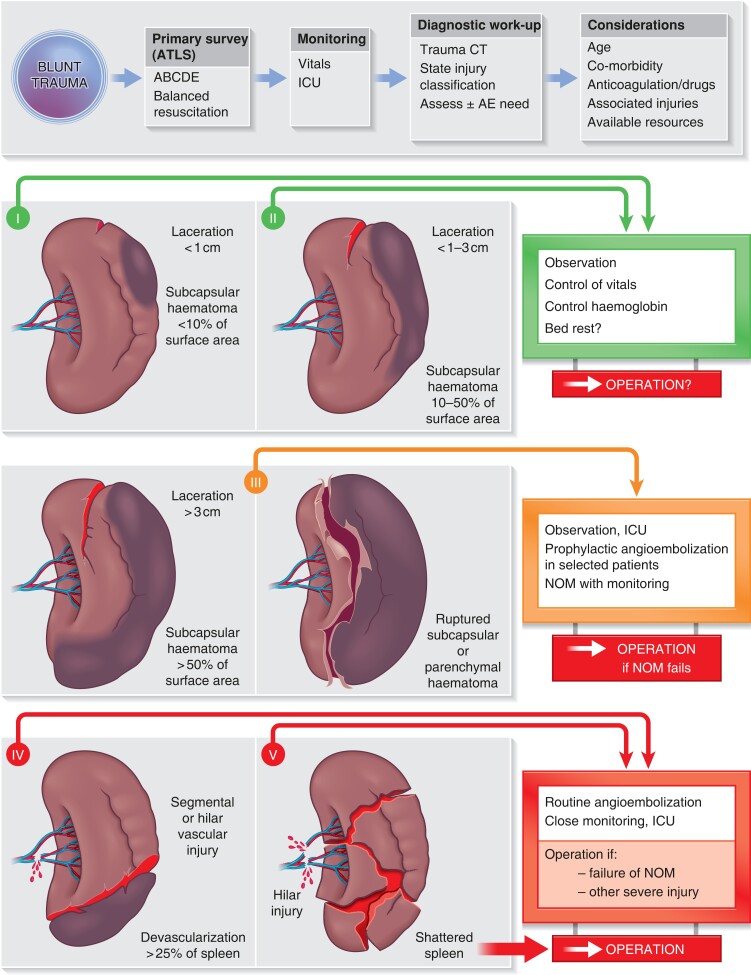
Management considerations in splenic injury after blunt trauma ATLS, Advanced Trauma Life Support; AE, angioembolization; NOM, non-operative management.

The severity of injury is classified by the extent of disruption of the splenic anatomy, as described by the American Association for the Surgery of Trauma (AAST) Organ Injury Scale^2^. As the vast majority of splenic injuries (80–90 per cent) undergo non-operative management, severity grades (*Fig. 1*) are usually based on imaging (that is, a trauma protocol with contrast-enhanced CT). Inter-rater and intrarater variability is less than perfect among radiologists, potentially leading to variation in management for a number of patients^3,4^, as exemplified by the Nijmegen consensus process on grade III injuries^5^. CT criteria define vascular injury as pseudoaneurysm, arteriovenous fistula or a contrast ‘blush’, all of which may indicate high-grade injury (AAST grade IV or V) (*Fig. 1*). Another scoring tool that incorporates the patient’s haemodynamic status has been proposed by the World Society of Emergency Surgery (WSES)^6^. Both classifications are applicable to adults and children, provided that age-appropriate criteria for haemodynamic instability are used in the WSES grading.

## Treatment options

Treatment should be tailored to age, presence of co-morbidities, and (changes in) physiological status of the patient (*Fig. 1*). Most centres undertake initial diagnostics and resuscitation in the emergency room (including chest and pelvic X-ray, and bedside ultrasonography) and proceed to cross-sectional imaging (protocol-based trauma imaging with CT and contrast-enhanced multiphase protocols according to need). Initial resuscitation should be done in parallel with monitoring of vital signs with observation, and preferably in an ICU or high-dependency ward, until definitive imaging and reporting has been completed and a care plan agreed. Patients who are haemodynamically unstable (for example, with severe hypotension, tachycardia, loss of consciousness) with no response to resuscitation should be taken to the operating theatre for trauma laparotomy. In centres with the availability, a hybrid suite that will allow simultaneous resuscitation, interventional diagnostics/therapeutics, and open surgery is the preferred place to be.

For patients with stable haemodynamics or adequate response to fluids, immediate diagnostic work-up should commence with cross-sectional imaging (CT with intravenous contrast in 3 phases). Further decision-making depends on patient factors, and the presence of other associated injuries that may or may not need surgery or intervention (*Fig. 1*).

Non-operative management should be preferred, irrespective of the grade of injury in patients of all ages, in the absence of other abdominal injuries requiring interventions, provided that the haemodynamic status permits a trial of non-operative management^6–8^. This requires close cooperation between all team members (surgeon, anaesthetist, intensivist, interventional radiologist, nurse staff, and coordinators) and intermittent reassessment of the patient. Age above 55 years, polytrauma with a high burden of injury, and moderate-to-high splenic injury are associated with failure of non-operative management. These patients warrant extra vigilance to change in physiology, with a low threshold for surgery. Anticoagulative/antiplatelet treatment is another obvious risk factor, and adequate transfusion and antidote measures might need to be taken.

Angioembolization is part of non-operative management, regardless of injury grade, to stop the bleeding or prevent rebleeding (blush on CT or formation of pseudoaneurysm/arteriovenous fistula). Angioembolization can be used for adults and children^6^, but failure of non-operative management increases with higher injury severity; the failure rate is up to 50 per cent in those with grade V injury. In adults, any vascular abnormalities or signs of bleeding should be sought actively with interventional angiography in grade IV and V injuries, with subsequent angioembolization recommended being either therapeutic or prophylactic. In children, the literature advocates a ‘less is more’ approach, reserving angioembolization for patients showing signs of continuous bleeding^9^ or for transient response to resuscitation^10^.

### What type of angioembolization is preferred for splenic injury?

Proximal splenic artery embolization decreases the perfusion pressure in the spleen. The viability of the splenic tissue will be maintained via collateral flow. Distal embolization causes segmental ischaemia and can be used to address focal injury. The two approaches seem comparable in efficacy and in recurrence of bleeding, but proximal embolization might lead to fewer complications^11,12^. Embolization will inevitably lead to a certain degree of ischaemia and possible necrotic tissue, which in some instances is complicated by the formation of a splenic abscess. This can be managed by antibiotics paired with percutaneous drainage as first-line treatment, and splenectomy when this fails^13^.

## When to operate?

Operative management of splenic injuries can be regarded as the last resort in stopping any non-critical bleeding, but may be the first choice in any grade of injury. Patient-related factors (age, co-morbidities, drugs), associated injuries, and available resources (hospital setting) all play a role in the decision to opt for conservative or operative management. If for any reason laparotomy is indicated, most of the spleen-preserving measures (for example, local haemostatic agents, packing, splenorrhaphy) can be applied if the state of the splenic tissue and the patient’s physiological status permit. Needing a laparotomy in the first place should prompt a definitive solution to the problem, making splenectomy the preferred treatment when facing relevant bleeding.

## Role of rescan in non-operative management

Repeat imaging in asymptomatic patients rarely results in further interventions, and the majority of vascular abnormalities are found in grade III injuries and higher. On this basis, recent recommendations suggest that, for adults, rescanning with contrast-enhanced ultrasonography (CEUS) or CT should be based on clinical findings for low-grade injuries and be mandatory for high-grade injuries 48–72 h after admission, irrespective of embolization status^14^. Notably, CEUS is not available universally (only in Europe and Asia). Children rarely need repeated imaging unless symptomatic, regardless of injury grade.

## Role of prophylactic splenic artery embolization?

A French multicentre RCT set out to determine whether prophylactic splenic artery embolization to reduce the risk of splenectomy was comparable or better than surveillance with embolization on demand for high-grade splenic injuries^15^. In this SPLASH (Splenic Arterial Embolization to Avoid Splenectomy) trial, both strategies resulted in a splenic rescue rate of more than 93 per cent, but many patients in the surveillance group received embolization within a few days after injury (cross-over between groups). The investigators concluded that, with follow-up including CT, surveillance with embolization on demand was acceptable practice. A supporting commentary addressed the need to take the environment for surveillance or treatment into account; for example, patients and providers may prefer angioembolization for those with long travel distances to a tertiary-care facility^16^.

## When to start prophylactic antithrombotic therapy after splenic injury in blunt trauma

Following trauma-induced coagulopathy, patients are at increased risk of venous thromboembolism. Studies using thromboelastography to examine the coagulation status have shown that this risk increases from 48 h after trauma. This leaves room for the administration of low molecular weight heparin (LMWH) to prevent thromboembolic events. Recent consensus states that LMWH should be initiated ‘within 24 h from hospital admission for patients with WSES class I (AAST grades I–II) and within 48–72 h for those with WSES class II–III (AAST grades III–V) splenic injuries’^14^. Updated guidelines from the Western Trauma Association^17^ on prophylactic antithrombotic therapy in injured patients include a helpful clinical algorithm. In patients with solid organ injury, these guidelines go even further, recommending administering LMWH within 24 h.

## Who needs vaccination?

Postsplenectomy vaccination against encapsulated bacteria and seasonal flu (risk of secondary bacterial infection) is recommended^18^. People without splenic function (asplenic or postsplenectomy state) have a greater than 50 times higher risk of overwhelming postsplenectomy infections, which may have a fatal course. However, the routine use of lifelong prophylactic antibiotics, practised in some countries for the prevention of overwhelming postsplenectomy infections, is not evidence-based^19^ and should not be recommended.

Recent practice management guidelines^20^ do not recommend routine vaccination after angioembolization. In this setting, retained splenic immune function has been demonstrated, with no data to suggest an increased level of infectious complications^20–22^.

## For how long should patients with splenic injury be restricted physically?

Historically, bed rest in the initial phase has been emphasized owing to the belief that movement, falling or even spikes in BP could disrupt clots leading to delayed bleeding. On the other hand, the pitfalls of prolonged bed rest include increased risk of deep vein thrombosis and thromboembolism, pneumonia, hospital infections, and increased duration of hospital stay and thereby increased costs.

Current recommendations allow early mobilization within 24 h for patients with low-grade splenic injury, and up to 2 days for those with high-grade injuries with stable clinical parameters and repeated, stable haemoglobin levels^14^. For children, no bed rest for AAST grade I, 1 night of bed rest for AAST grade II, and 2 nights is suggested for AAST grade III or higher when clinical parameters remain stable.

The duration of activity restriction, minimizing hard physical activity, heavy lifting, and contact sports, has shown a similar trend, with earlier return to preinjury activity level. Some 3–5 weeks for AAST grades I–II, and up to 2–4 months for AAST grades III–V have been proposed for adults. In children, major activity restrictions could be limited to 4 weeks after the injury, irrespective of injury grade on CT^14^.

## Summary

Taken together, for patients with isolated blunt splenic injury, a very high rate of successful non-operative, spleen-salvaging management can be expected with use of selective angioembolization. For patients with considerable co-morbidity, older age, and particularly those with other relevant organ injuries from blunt trauma, the success rate may be lower. In the setting of more complex combinations of factors to consider, the use of prophylactic angioembolization, the need for ongoing surveillance, and need for change in management as a response to altered physiological parameters must be considered within an ongoing team discussion. Splenectomy should be the safe choice where spleen salvage is unlikely to succeed or is felt to be of an unwarranted high risk.
